# Association of Accelerometer-Measured Light-Intensity Physical Activity With Brain Volume: The Framingham Heart Study

**DOI:** 10.1001/jamanetworkopen.2019.2745

**Published:** 2019-04-19

**Authors:** Nicole L. Spartano, Kendra L. Davis-Plourde, Jayandra J. Himali, Charlotte Andersson, Matthew P. Pase, Pauline Maillard, Charles DeCarli, Joanne M. Murabito, Alexa S. Beiser, Ramachandran S. Vasan, Sudha Seshadri

**Affiliations:** 1 Department of Endocrinology, Diabetes, Nutrition and Weight Management, Boston University School of Medicine, Boston, Massachusetts; 2 Framingham Heart Study, Framingham, Massachusetts; 3 Department of Biostatistics, Boston University School of Public Health, Boston, Massachusetts; 4 Department of Neurology, Boston University School of Medicine, Boston, Massachusetts; 5 Department of Internal Medicine, Glostrup Hospital, Glostrup, Denmark; 6 Melbourne Dementia Research Centre, The Florey Institute for Neuroscience and Mental Health, Melbourne, Victoria, Australia; 7 Faculty of Medicine, Dentistry, and Health Sciences, The University of Melbourne, Melbourne, Victoria, Australia; 8 Centre for Human Psychopharmacology, Swinburne University of Technology, Melbourne, Victoria, Australia; 9 Department of Neurology and Center for Neuroscience, University of California, Davis; 10 Department of Medicine, Boston University School of Medicine, Boston, Massachusetts; 11 Department of Epidemiology, Boston University School of Public Health, Boston, Massachusetts; 12 Glenn Biggs Institute for Alzheimer’s and Neurodegenerative Diseases, University of Texas Health Sciences Center, San Antonio

## Abstract

**Importance:**

Dementia risk may be attenuated by physical activity (PA); however, the specific activity levels optimal for dementia prevention are unclear. Moreover, most older adults are unable to meet the nationally recommended PA guidelines, set at 150 minutes of moderate to vigorous PA per week.

**Objective:**

To assess the association of total steps walked per day and total dose (intensity × duration) of PA with brain volumes on magnetic resonance imaging (MRI) among Framingham Heart Study participants.

**Design, Setting, and Participants:**

This cross-sectional, community-based cohort study of the association of accelerometry-determined PA with brain MRI measures in Framingham, Massachusetts, included the Framingham Heart Study third-generation (examination 2, 2008-2011) and offspring (examination 9, 2011-2014) cohorts. Of 4021 participants who agreed to wear an accelerometer and had valid data (≥10 hours/day for ≥3 days), 1667 participants who did not undergo brain MRI (n = 1604) or had prevalent dementia or stroke (n = 63) were excluded. Data analysis began in 2016 and was completed in February 2019.

**Exposures:**

Physical activity achieved using accelerometry-derived total activity (steps per day) and 2 intensity levels (light intensity and moderate to vigorous intensity).

**Main Outcomes and Measures:**

Differences in total brain volume and other MRI markers of brain aging.

**Results:**

The study sample of 2354 participants had a mean (SD) age of 53 (13) years, 1276 (54.2%) were women, and 1099 (46.7%) met the PA guidelines. Incremental light-intensity PA was associated with higher total brain volume; each additional hour of light-intensity PA was associated with approximately 1.1 years less brain aging (β estimate, 0.22; SD, 0.07; *P* = .003). Among individuals not meeting the PA guidelines, each hour of light-intensity PA (β estimate, 0.28; SD, 0.11; *P* = .01) and achieving 7500 steps or more per day (β estimate, 0.44; SD, 0.18; *P* = .02) were associated with higher total brain volume, equivalent to approximately 1.4 to 2.2 years less brain aging. After adjusting for light-intensity PA, neither increasing moderate to vigorous PA levels nor meeting the threshold moderate to vigorous PA level recommended by the PA guidelines were significantly associated with total brain volume.

**Conclusions and Relevance:**

Every additional hour of light-intensity PA was associated with higher brain volumes, even among individuals not meeting current PA guidelines. These data are consistent with the notion that the potential benefits of PA on brain aging may accrue at a lower, more achievable level of intensity or duration.

## Key Points

**Question:**

Is light-intensity physical activity associated with brain volume?

**Findings:**

In this community-based cohort study of 2354 participants, each additional hour spent in light-intensity physical activity was associated with larger brain volume, equivalent to approximately 1.1 years less brain aging. Achieving 10 000 or more steps per day was associated with higher brain volume compared with those achieving fewer than 5000 steps per day.

**Meaning:**

Incremental physical activity, even at a more practical light intensity, may be involved in the maintenance of brain structures into older age.

## Teaser

This community-based cohort study investigates the association of total steps walked per day and total intensity and duration of physical activity with brain volumes on magnetic resonance imaging among Framingham Heart Study participants.

## Introduction

Considerable evidence suggests that engaging in regular physical activity (PA) may prevent cognitive decline and dementia.^1,2,3,4,5^ Active individuals have lower metabolic and vascular risk factors,^6^ and these risk factors may explain these individuals’ propensity for healthy brain aging.^7,8,9,10^ Even short-term exercise interventions have been shown to prevent hippocampal atrophy in older adults^11^ and may also improve brain connectivity.^12^ Furthermore, cross-sectional epidemiologic studies have established an association of physical inactivity with brain aging.^4,13,14,15,16,17,18^ However, further work is needed to pinpoint the optimal dosage (duration × intensity) of PA needed to promote healthy brain aging. Although this question cannot be fully addressed in an observational study, our investigation sheds light on the dosage of PA most strongly associated with favorable brain structure in a community setting.

The new US Department of Health and Human Services (HHS) 2018 Physical Activity Guidelines for Americans^19^ suggest that some PA is better than none, but achieving 150 or more minutes of moderate to vigorous physical activity (MVPA) per week is recommended for substantial health benefits. For the first time, the PA guidelines now include brain health in the list of benefits.^19,20^ Because the estimated proportion of US residents meeting the level of MVPA recommended by the guidelines decreases sharply from middle age (57% of individuals aged 40-49 years) to older age (26% of those aged 60-69 years),^21^ we assessed whether the association of PA with healthy brain aging extends to intensities and durations of PA below the threshold of PA dosage recommended by the guidelines.

## Methods

We assessed PA using Actical accelerometers (model 198-0200-00; Philips Respironics) in 4334 participants from the Framingham Heart Study third-generation (examination 2, 2008-2011) and offspring (examination 9, 2011-2014) cohorts who attended our study site at this examination (ie, were not seen off-site) and agreed to wear the devices,^22,23^ from a total of 5841 who participated in these examinations (eFigure in the Supplement). Brain magnetic resonance imaging (MRI) assessments were completed from June 2009 to December 2014. Participants were excluded if they wore the accelerometer for less than 3 valid days (ie, ≥10 h/d; n = 313), did not undergo brain MRI (n = 1604), or had dementia, stroke, or another disorder that may affect brain MRI measures (n = 63). The final sample size used for the main analysis was 2354 participants. Data analysis began in 2016 and was completed in February 2019. The institutional review board from Boston University School of Medicine approved the study. All participants provided written informed consent. This article follows the Strengthening the Reporting of Observational Studies in Epidemiology (STROBE) reporting guideline for cohort studies.

### Physical Activity Accelerometry

Total PA (steps per day), MVPA, light-intensity PA, and sedentary time were measured using an Actical accelerometer. The devices were worn on a belt attached to the hip for 8 days, but at least 3 days of data were required for inclusion in our analyses.^24^ Participants in the third-generation cohort were instructed to wear the accelerometer 24 hours per day. We encountered difficulty distinguishing between sedentary and sleep time; hence, at the later offspring cohort examination cycle, we instructed participants to wear the accelerometer during waking hours only. All participants were instructed to remove the device for bathing or swimming. This device recorded signals within 0.5 to 3 Hz and accelerations and decelerations within 0.05 to 2 *g*. Recorded signals were grouped into counts or steps, averaged over 1-minute intervals, and analyzed using a customized software (KineSoft version 3.3.63 [KineSoft]), with validation data reported previously.^25^

To partially account for differences in wear time between the 2 cohorts, activity measures such as sedentary time (<200 counts/min) and light-intensity PA (200-1486 counts/min) were reported as a percentage of wear time during the 16 hours between 6 am and 10 pm and standardized to a 16-hour day. Moderate to vigorous PA (defined as >1486 counts/min) and total steps per day were considered any time (midnight-11:59 pm) and were reported in minutes of MVPA and number of steps instead of in relation to wear time. Data accumulated on the first day the device was worn were not included in data processing because it was only a partial day, starting after the examination at the research center. Additionally, nonwear time (defined as 60 minutes of 0 counts, with 2 minutes of interruptions allowed) was also removed from the total wear time and, therefore, was not included for the accumulation of PA or sedentary time data.

### Brain MRI Imaging

Brain MRI images were obtained on a Siemens 1.5-T scanner (Siemens Medical Solutions) using T1-weighted coronal spoiled gradient-recalled echo acquisition and fluid-attenuated inversion recovery sequences with standard MRI parameters. Our primary outcome was total cerebral brain volume (TCBV). Secondary outcomes included hippocampal volume, white matter hyperintensities volume, total white matter volume, total gray matter volume, cortical gray matter volume, and lateral ventricular volume. Complete information on the MRI procedures of the Framingham Heart Study can be seen elsewhere.^26^ All volumes were corrected for differences in head size by computing these variables as percentages of total cranial volume.^26,27,28,29^ In addition, white matter hyperintensities volumes were log-transformed to normalize population variance. The number of years of brain aging associated with specific PA levels was estimated by using the reported association of age with TCBV (0.2% TCBV loss per year of aging).^10^

### Statistical Analysis

We first compared brain volumes between individuals who did or did not meet the current PA guidelines (defined as averaging ≥21.4 minutes of MVPA per day, equivalent to ≥150 min/wk). Means were adjusted for age, age squared, time between examination and MRI, sex, cohort, smoking status, season during which the examination was conducted (examination season), residence in New England vs other regions, wear time, body mass index, stage 2 hypertension (HTN, using the 2017 American College of Cardiology and American Heart Association Hypertension Guideline of systolic blood pressure ≥140 mm Hg or diastolic blood pressure ≥90 mm Hg),^30^ diabetes, and cardiovascular disease (CVD) (eTable 1 in the Supplement). Next, we used multivariable regression models to find the association of PA levels (independent variable) with brain volumes (dependent variable), adjusting for age, age squared, sex, cohort, smoking status, time from examination to MRI, examination season, residence in New England vs other, and wear time. In secondary regression models, we additionally adjusted for vascular risk factors (body mass index, HTN, diabetes, and CVD) (eTable 1 in the Supplement).

The number of steps walked per day represented a metric of global PA and, therefore, was modeled separately from other PA variables. Light-intensity PA and MVPA were each modeled separately and together in a single model in association with brain MRI measures. We were interested in understanding which PA intensity level was the more predominant factor in association with brain aging, despite the previously reported modest association of MVPA with light-intensity PA in the Framingham offspring cohort (*r* = 0.30; *P* < .001).^31^ All analyses were performed in the full sample and in 2 subsets of the population that had smaller MVPA achievement (an average of <21.4 min/d MVPA and <10 min/d MVPA, respectively) to observe whether incremental PA (light-intensity PA and steps) were associated with brain MRI measures. Results were not reported separately for sedentary time because it was strongly correlated with light-intensity PA (*r* = −0.90; *P* < .001).^31^ The addition of sedentary time to light-intensity PA time makes up almost 98.7% of total wear time for individuals not meeting the guidelines or 97.0% of wear time for all participants (Table 1). Steps (<5000, 5000-7499, 7500-9999, and ≥10 000 steps/d) and MVPA (<10, 10-19, 20-29, and ≥30 min/d) were modeled categorically, owing in part to skewed distributions, while light-intensity PA was modeled linearly. Association estimates from regression models were considered significant at a *P *value less than .05; tests were 2-tailed. Interactions by age were also tested. Despite interaction tests producing *P* values greater than .10, we stratified by age group to evaluate any differences in the shape of association of PA measures with TCBV.

**Table 1.** Demographic and Health Characteristics, Physical Activity, and MRI Measures of 2354 Participants

Variable	Total (N = 2354)	Not Meeting PA Guidelines (n = 1255)	Meeting PA Guidelines (n = 1099)
**Demographic Characteristics**			
Age, mean (SD), y	53 (13)	56 (14)	49 (11)
Female, No. (%)	1276 (54.2)	754 (60.1)	522 (47.5)
Offspring cohort, No. (%)	1691 (71.8)	470 (37.5)	193 (17.6)
Time to MRI, mean (SD), y	1.6 (0.9)	1.6 (0.9)	1.6 (0.9)
Education, No. (%)			
<High school degree	10 (0.4)	8 (0.6)	2 (0.2)
High school degree	547 (23.2)	377 (30.0)	170 (15.5)
Some college	501 (21.2)	285 (22.7)	216 (19.7)
College degree	1291 (54.8)	583 (46.5)	708 (64.4)
New England residence, No. (%)	2084 (88.5)	1120 (89.2)	964 (87.7)
**Health Characteristics, No. (%)**			
Current smoker	152 (6.5)	93 (7.4)	59 (5.3)
Stage 2 hypertension^a^	739 (31.4)	497 (39.6)	242 (22.0)
History of diabetes	143 (6.1)	117 (9.3)	26 (2.4)
History of cardiovascular disease	98 (4.1)	72 (5.7)	26 (2.4)
**Physical Activity**			
Steps/d, median (IQR)	7519 (5419-10 059)	5820 (4234-7689)	9582 (7646-12 304)
MVPA, median (IQR), min/d	19.9 (9.8-34.9)	10.3 (5.4-15.8)	36.3 (27.9-49.6)
Wear time, mean (SD), min	883 (111)	853 (109)	917 (104)
Light-intensity PA, mean (SD), % wear time	14.8 (5.2)	13.6 (4.9)	16.2 (5.3)
Light-intensity PA, mean (SD), h per standardized 16-h day	2.37 (0.83)	2.18 (0.78)	2.59 (0.85)
Sedentary time, mean (SD), % wear time	82.2 (6.4)	85.1 (5.2)	78.9 (6.0)
Sedentary time, mean (SD), h per standardized 16-h day	13.15 (1.02)	13.62 (0.83)	12.62 (0.96)
**MRI Measures**^b^			
Total cerebral brain volume, mean (SD), %	87.3 (3.7)	86.5 (3.9)	88.1 (3.2)
Hippocampal volume, mean (SD), %	0.5 (0.05)	0.5 (0.05)	0.5 (0.04)
Lateral ventricular volume, mean (SD), %	1.4 (1.1)	1.6 (1.2)	1.2 (0.9)
White matter hyperintensities volume, median (IQR), %	0.05 (0.02-0.14)	0.07 (0.03-0.21)	0.04 (0.02-0.08)
White matter hyperintensities volume, mean (SD), log of %	−2.87 (1.42)	−2.56(1.46)	−3.23 (1.28)
Total white matter volume, mean (SD), %	40 (2)	40 (2)	40 (2)
Total gray matter volume, mean (SD), %	49 (2)	49 (2)	50 (2)
Cortical gray matter volume, mean (SD), %	38 (2)	37(2)	38 (2)

Abbreviations: IQR, interquartile range; MRI, magnetic resonance imaging; MVPA, moderate to vigorous physical activity; PA, physical activity.

^a^Stage 2 hypertension was defined using the 2017 American College of Cardiology/American Heart Association Hypertension Guideline of systolic blood pressure 140 mm Hg or higher or diastolic blood pressure 90 mm Hg or higher.^30^

^b^There were no differences in adjusted means for brain MRI measures among those meeting and not meeting the guidelines after adjusting for age, age squared, sex, time from examination to MRI, smoking status, cohort, wear time, season of examination, residence in New England, body mass index, hypertension, diabetes, and cardiovascular disease (eTable 1 in the Supplement).

## Results

### PA and Brain Volume in the Full Study Sample

In our final sample of 2354 participants, the mean (SD) age was 53 (13) years, and 1276 (54.2%) were women (Table 1). A total of 1099 participants (46.7%) met the PA guidelines. Among those meeting the guidelines, fewer participants had HTN (242 [22.0%]), diabetes (26 [2.4%]), and CVD (26 [2.4%]) than those not meeting the guidelines (497 [39.6%] had HTN, 117 [9.3%] had diabetes, and 72 [5.7%] had CVD). Brain measures were also found to be different between persons who did and did not meet the guidelines (Table 1), but these differences did not remain significant after adjustments for age and other covariates (eTable 1 in the Supplement). However, taking more steps per day or participating in more light-intensity activity was associated with larger TCBV (Table 2). Total cerebral brain volume declines at a rate of 0.2% per year after age 60 years.^10^ Therefore, compared with persons who averaged less than 5000 steps/d, achieving 10 000 steps/d or more was associated with approximately 1.75 years less brain aging (approximately 0.35% higher brain volume). Each additional hour spent in light-intensity PA was associated with approximately 1.1 years less brain aging (approximately 0.22% higher brain volume; β estimate, 0.22; SD, 0.07; *P* = .003) (Table 2). Also, achieving just 10 to 19 minutes of MVPA, compared with less than 10 minutes MVPA, was associated with larger TBCV. However, after adjusting for light-intensity PA, increasing MVPA levels was not significantly associated with TCBV. Although provided for context, the approximations provided in years of brain aging should be viewed cautiously as the participants’ mean age was younger than 60 years.

**Table 2.** Association of Objectively Measured Physical Activity With Brain Magnetic Resonance Imaging Measures in the Full Study Sample of 2354 Participants

Category of PA	No. of Participants	Model 1: Total Cerebral Brain Volume^a^		Model 2: Total Cerebral Brain Volume, Including Light-Intensity PA and MVPA^b^	
		β Estimate (SE)	*P *Value	β Estimate (SE)	*P *Value
MVPA, min/d					
<10	602	0	1 [Reference]	0	1 [Reference]
10-19	580	0.36 (0.16)	.03	0.29 (0.16)	.08
20-29	421	0.43 (0.18)	.02	0.35 (0.18)	.06
≥30	751	0.24 (0.16)	.14	0.14 (0.17)	.39
Light-intensity PA, per 1-h increment	NA	0.24 (0.07)	.001	0.22 (0.07)	.003
Total activity, steps/d					
<5000	489	0	1 [Reference]	NA	NA
5000-7499	684	0.09 (0.16)	.60	NA	NA
7500-9999	581	0.29 (0.17)	.10	NA	NA
≥10 000	596	0.35 (0.18)	.05	NA	NA

Abbreviations: MVPA, moderate to vigorous physical activity; NA, not applicable; PA, physical activity.

^a^ Adjusted for age, age squared, sex, time from examination to magnetic resonance imaging, smoking status, cohort, wear time, season of examination, and residence in New England.

^b^ Light-intensity PA models were additionally adjusted for MVPA categories (≥10 min vs <10 min); MVPA models were additionally adjusted for light-intensity PA. Additional models (eTable 2 in the Supplement), adjusted for body mass index, hypertension, diabetes, and cardiovascular disease, had no effect on significance for any analysis reported and had only minor impact on effect sizes.

### PA and Brain Volume in Persons Not Meeting PA Guidelines

Among participants not achieving the PA guidelines, incremental light-intensity PA was associated with higher TCBV, so that every additional hour of light-intensity PA was associated with approximately 1.4 years less brain aging (per hour increment: β estimate, 0.28; SD, 0.11; *P* = .01) (Table 3). Also, individuals performing 7500 steps or more per day had larger brain volumes, equivalent to approximately 2.2 years less brain aging compared with those achieving less than 7500 steps per day (β estimate, 0.44; SD, 0.18, *P* = .02). The threshold of 7500 steps per day was chosen for this subanalysis because it appeared to be the threshold at which effect sizes (β estimates) for steps per day were associated with TCBV in the full study sample.

**Table 3.** Association of Objectively Measured Physical Activity With Brain Magnetic Resonance Imaging Measures in Subsamples of Individuals Not Meeting PA Guidelines

PA Measures	Subsample A: Individuals with MVPA <21.4 min/d (n = 1255)^a^		Subsample B: Individuals with MVPA <10 min/d (n = 602)^b^	
	β Estimate (SE)^c^	*P *Value	β Estimate (SE)^c^	*P *Value
Light-intensity PA,^d^ per 1-h increment	0.28 (0.11)	.01	0.41 (0.18)	.03
Total activity ≥7500 steps/d vs <7500 steps/d^e^	0.44 (0.18)	.02	0.32 (0.34)	.35

Abbreviations: PA, physical activity; MVPA, moderate to vigorous physical activity.

^a^ Not meeting the PA guidelines, with a mean of less than 21.4 min/d of MVPA or less than 150 min/wk of MVPA.

^b^ Not meeting the PA guidelines, with a mean of less than 10 min/d of MVPA or less than 70 min/wk of MVPA.

^c^ Model adjusted for age, age squared, sex, time from examination to MRI, smoking status, cohort, wear time, season of examination, and residence in New England. Light-intensity PA models were additionally adjusted for MVPA.

^d^ For subsample A, adjusted for MVPA (≥10 min/d vs <10 min/d); for subsample B, adjusted for MVPA (minutes per day).

^e^ For subsample A, 333 participants achieved 7500 steps/d or more, and 918 achieved less than 7500 steps/d. For subsample B, 93 participants achieved 7500 steps/d or more, and 506 achieved less than 7500 steps/d.

### Sensitivity Analysis

Significant associations of objective measures of PA with TCBV remained after excluding 271 participants with prevalent CVD or self-reported functional limitations that prevented them from walking 1 block or climbing 1 flight of stairs (data not shown). These associations also remained after adjustment for vascular risk factors (body mass index, HTN, diabetes, and CVD) (eTable 2 in the Supplement). Furthermore, we stratified our results by decade and observed that the trends for associations of PA measures with TCBV were present in participants in their 50s, 60s, and 70s, although not observed in participants in their 40s (Figure; eTable 3 in the Supplement).

**Figure.**
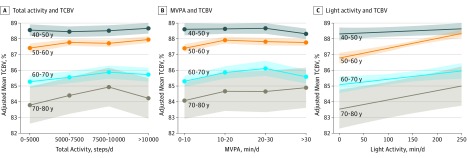
Association of Physical Activity With Total Cerebral Brain Volume (TCBV) by Age Category Shaded areas indicate SE bars; MVPA, moderate to vigorous physical activity. A, Adjusted for age, age squared, time between examination and magentic resonance imaging (MRI), sex, cohort, smoking status, examination season, residence in New England, and wear time. B, Adjusted for age, age squared, time between examination and MRI, sex, cohort, smoking status, examination season, residence in New England, wear time, and light-intensity activity. C, Adjusted for age, age squared, time between examination and MRI, sex, cohort, smoking status, examination season, residence in New England, wear time, and MVPA.

### PA and Regional Brain Volumes

In the full study sample, steps per day and light-intensity PA were associated with smaller lateral ventricles, corroborating our findings with TCBV (eTable 2 in the Supplement). Additionally, achieving just 10 to 19 min/d of MVPA, compared with less than 10 min/d of MVPA, was associated with higher total white matter volume. Surprisingly, we also observed that more light-intensity PA was associated with higher white matter hyperintensities volume, an index of brain injury. However, no associations with hippocampal volume or gray matter volume were observed (eTable 4 in the Supplement).

## Discussion

The HHS released an update to its PA guidelines in 2018, suggesting that some PA is better than none, while continuing to promote the achievement of 150 min/wk of MVPA (equivalent to approximately 21.4 min/d, on average) for substantial health benefits.^19^ The MVPA recommendation has been the focus of previous guidelines from the HHS^32^ and other organizations, including the American College of Sports Medicine, American Heart Association,^33^ and the World Health Organization.^34^ Our observations support this new update to the guidelines, suggesting that incremental PA was associated with larger total brain volume at a relatively low intensity threshold (as measured by light-intensity PA). However, our observation that MVPA was not significantly associated with brain volume after adjusting for light-intensity PA suggests that it is unclear whether individuals can expect further gain in benefit with higher-intensity activity. Because our study sample had higher average PA levels (with 1099 participants [46.7%] meeting PA guidelines) than US population-based study samples of comparable age ranges (on average, 39% of adults aged 50-59 years meet the guidelines),^21,35^ the implications of physical inactivity associated with lower brain volume may have an even larger impact in the general US population.

There has been a growing body of literature establishing light-intensity PA as an important factor for improving health outcomes,^36^ but in our review of the literature, light-intensity PA has not often been considered separately from total PA for its association with brain structure. Previous studies have identified positive associations of self-reported PA with brain volume,^4,13^ but accelerometry studies often have smaller sample sizes and have focused on examining the association of total PA with brain volume.^14,15,16,17,18^ To our knowledge, only 1 previous study in older Japanese adults with mild cognitive impairment observed that MVPA, but not light-intensity PA, was associated with larger brain volume.^15^ However, PA variables are associated with one another, so in our analyses, we went a step further and modeled them together to determine what type of PA intensity (low or high) is driving the association of PA with brain volume. Other studies have reported that total PA, but not higher-intensity PA or sedentary behavior, was associated with brain white matter volume and integrity.^14,18^ However, not all significant findings have been focused on the brain’s white matter structure. Two 2018 studies have linked total PA with brain gray matter volume but not white matter volume or the total cortex volume.^16,17^ It may be possible that PA plays a role in maintaining the integrity of multiple brain subregions.

The simplification of PA as a predictor variable has potentially masked more nuanced associations of components of PA with brain health. Compared with previous research, our study provides multiple PA levels and intensities and uses accelerometry-determined intensity thresholds (ie, light-intensity PA and MVPA) in the same statistical models to provide a more sensitive measure of PA doses and examine what type of PA is driving the associations we observe.

To our knowledge, our study sample was, on average, the youngest of studies assessing objectively measured PA and brain structure. Therefore, it was important to use our data set to determine whether these associations differ by age strata. Our observed association of low PA with smaller brain volume even in middle age (≥50 years) is consistent with the notion that some adults may enter older age with lower brain volumes, putting them at a disadvantage for maintaining this already depleted tissue. Furthermore, differences in the size of the association of PA with brain volume in participants 50 years or younger may be owing to less variation in brain volumes in the younger sample because brain atrophy dramatically increases during individuals’ 50s and 60s.^10,26^ Our results are consistent with a study in the UK Biobank that observed significant associations only in participants younger than 60 years.^17^

The association of PA with vascular risk factors^37^ is frequently cited as a mechanism responsible for the link between PA and dementia risk.^38^ We previously reported that lower fitness levels and suboptimal hemodynamic response to exercise in middle-aged Framingham Heart Study offspring participants were associated with smaller brain volumes 2 decades later.^39^ Lower total brain volumes, increased white matter hyperintensities volume, and enlarged lateral ventricles have been associated with elevated vascular risk factors previously and play a role in cognitive decline.^7,10,40^ In the current study, individuals achieving less than 5000 steps/d or less than 10 min/d of MVPA (compared with 20-29 min/d of MVPA) were more likely to have enlarged lateral ventricles. These associations remained statistically significant even after adjusting for prevalent CVD and common vascular risk factors. However, it is still possible that vascular mechanisms are involved in the observed association of PA with brain volume.

Results from our study also suggest that part of the association of PA with brain volume may be driven by maintenance of total white matter volume. Cerebral white matter is made up of the myelinated axons connecting the neurons that make up gray matter.^41^ The integrity of this myelin begins to slowly deteriorate in middle age, with an acceleration in older age, making up much of brain matter loss.^42^ Complex tasks that link brain regions, such as visuomotor control or learning a new skill, have been associated with increases in white matter microstructure, as reflected by changes in white matter fractional anisotropy measured following a 6-week intervention in which adults were taught how to juggle.^41,43^ Thus, PA may promote maintenance of white matter volume by implicating the visuomotor system. We observed a positive association of MVPA with total white matter volume in our study. Small observational studies (n < 100) have also demonstrated associations of accelerometer-determined PA with white matter integrity using fractional anisotropy in older adults.^14,44^

Our results are not in complete agreement with previous research suggesting increased PA is associated with preservation of hippocampal volume,^11^ as we observed no consistent association of PA with hippocampal volume. We also observed counterintuitive associations of higher light-intensity PA with greater vascular injury to the white matter as measured by white matter hyperintensities volumes. One important point to make about light-intensity PA is that it has a very strong negative association with sedentary time. Previous literature suggests that some sedentary activities may actually be beneficial to the brain when they involve cognitively complex tasks, such as computer use.^45^ For example, individuals with cognitively complex occupations that require long hours in front of a computer may achieve lower light-intensity PA but still have some protection against brain aging. Therefore, future research investigating the contexts and types of PA and sedentary time that influence brain aging will be very important.

### Limitations

We acknowledge that many of our significant findings may not hold up to multiple testing but may be hypothesis generating. Therefore, we presented them for completeness. However, the association of light-intensity PA with brain volume is consistent, regardless of adjustment model. The other results must be externally replicated for validation, especially in older age groups and longitudinal analyses. They must also be tested in an ethnically diverse population because the current cohort sample was predominantly white and of European descent.

Our study is cross-sectional, meaning that we cannot infer the temporal relationship of PA with brain volume. Although we hypothesize that higher PA may protect against brain atrophy through a number of mechanisms, including improved vascular health, brain atrophy may also lead to changes in PA. Cerebrovascular pathologies, including brain atrophy, are associated with changes in gait patterns.^46,47^ Results from the Cardiovascular Risk Factors, Aging and Dementia Study^18^ in 2016 demonstrated that baseline and 5-year change in brain volume were associated with lower PA at follow-up. The authors of this Finnish study observed decrements in PA and brain volume, which occurred in parallel, but causality remains unclear. In the secondary analysis of our study, we reported that excluding individuals who had CVD or reported a functional mobility limitation had no major impact on our results. To extend our findings, longitudinal studies with longer follow-up times are required to examine the role of PA and changes in gait patterns with respect to brain atrophy. Comorbidities, such as cardiovascular dysfunction, that could influence physical functioning^48^ and brain aging^49^ should also be studied in further analysis.

The public health significance of our study is in our further understanding of the potential value of light-intensity PA. Therefore, a practical requirement is to translate our accelerometer-derived definitions to estimates generated by other commonly worn devices, such as pedometers. Pedometers have been shown to produce lower daily step counts compared with accelerometer devices by approximately 2500 steps/d.^50,51,52^ Therefore, translating the absolute level of steps per day associated with brain MRI measures should be considered cautiously. We must also consider that the cut point we used to distinguish between light-intensity PA and MVPA was validated in young and middle-aged adults.^25^ Using a standard cut point across age and fitness levels may underestimate the metabolic equivalents achieved by our population, because older adults with poor fitness levels will achieve higher metabolic equivalent levels than individuals with higher fitness levels.^53^ However, our study design decision to use constant accelerometer cut points across age categories enables us to compare the pace (speed) of movement in our full study sample and minimizes misclassification of PA that could occur from selecting different intensity threshold cut points based on age.

## Conclusions

The results from our study suggest that the threshold of the favorable association for PA with brain aging may be at a lower, more achievable level of intensity or volume. Our investigation is in agreement with the HHS 2018 PA guidelines, which suggest that some PA is better than none. These data will need to be replicated in other cohorts and should be tested in intervention trials.

## Notes

Supplement. **eFigure.** Diagram illustrating derivation of analytic sample used for the main analysis **eTable 1.** Adjusted means of brain MRI measures, no significant differences between those meeting and not meeting the PA Guidelines **eTable 2.** Relations of objectively measured physical activity to brain MRI measures in the full study sample (n=2354), with 2 adjustment models **eTable 3.** Relations of objectively measured physical activity to total cerebral brain volume in participants aged 40-79 years (n=1922), by decade of age **eTable 4.** Relations of objectively measured physical activity to secondary brain MRI measures in the full study sample (n=2354), with 2 adjustment models
