# Error in Discussion

**DOI:** 10.1001/jamanetworkopen.2019.5153

**Published:** 2019-05-10

In the Original Investigation titled “Association of Accelerometer-Measured Light-Intensity Physical Activity With Brain Volume: The Framingham Heart Study,”^1^ published April 19, 2019, there was an error in the last sentence of the fourth paragraph of the Discussion. The sentence should read as follows: “Our results are consistent with a study in the UK Biobank that observed significant associations only in participants older than 60 years.” This article has been corrected.^1^
