# Supplementary material for: Association of Accelerometer-Measured Light-Intensity Physical Activity With Brain Volume: The Framingham Heart Study
Source: JAMA Netw Open. 2019 Apr 19;2(4):e192745. doi: 10.1001/jamanetworkopen.2019.2745 (PMC6481600; doi:10.1001/jamanetworkopen.2019.2745)
Supplement: Supplement. — eFigure. Diagram illustrating derivation of analytic sample used for the main analysis eTable 1. Adjusted means of brain MRI measures, no significant differences between those meeting and not meeting the PA Guidelines eTable 2. Relations of objectively measured physical activity to brain MRI measures in the full study sample (n=2354), with 2 adjustment models eTable 3. Relations of objectively measured physical activity to total cerebral brain volume in participants aged 40-79 years (n=1922), by decade of age eTable 4. Relations of objectively measured physical activity to secondary brain MRI measures in the full study sample (n=2354), with 2 adjustment models [file jamanetwopen-2-e192745-s001.pdf]

Supplementary Online Content

Spartano NL, Davis-Plourde KL, Himali JJ, et al. Association of accelerometer-measured light-intensity physical activity with brain volume: the Framingham Heart Study. *JAMA Netw Open.* 2019;2(4):e192745. doi:10.1001/jamanetworkopen.2019.2745

eFigure. Diagram illustrating derivation of analytic sample used for the main analysis
eTable 1. Adjusted means of brain MRI measures, no significant differences between those meeting and not meeting the PA Guidelines
eTable 2. Relations of objectively measured physical activity to brain MRI measures in the full study sample (n=2354), with 2 adjustment models
eTable 3. Relations of objectively measured physical activity to total cerebral brain volume in participants aged 40-79 years (n=1922), by decade of age
eTable 4. Relations of objectively measured physical activity to secondary brain MRI measures in the full study sample (n=2354), with 2 adjustment models

This supplementary material has been provided by the authors to give readers additional information about their work.

© 2019 Spartano NL et al. *JAMA Network Open.*

eFigure 1. Diagram illustrating derivation of analytic sample used for the main analysis

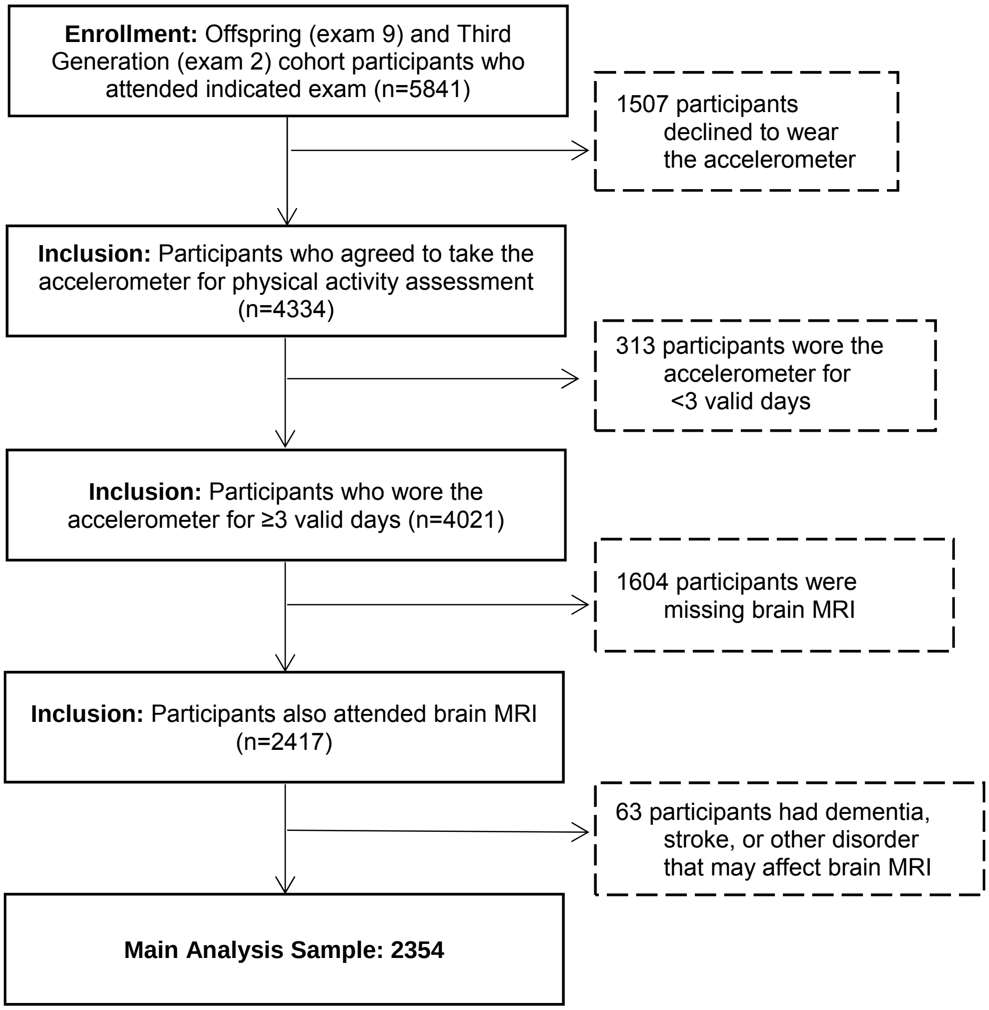

© 2019 Spartano NL et al. *JAMA Network Open.*

eTable 1. Adjusted means of brain MRI measures, no significant differences between those meeting and not meeting the PA Guidelines

	NOT meeting PA-guidelines (n=1255)	Meeting PA-guidelines (n=1099)	P for difference
**Total Cerebral Brain Volume, %** (mean±SE)	87.27± 0.079	87.34 ± 0.086	0.572
**Hippocampal Volume, %** (mean±SE)	0.54 ± 0.0013	0.54 ± 0.0015	0.194
**Lateral Ventricular Volume, %** (mean±SE)	1.46 ± 0.027	1.41 ± 0.029	0.299
**White Matter Hyperintensities Volume, log%** (mean±SE)	-2.86 ± 0.028	-2.90 ± 0.031	0.365
**Total white matter volume, %** (mean±SE)	40.21 ± 0.061	40.12 ± 0.065	0.291
**Total gray matter volume, %** (mean±SE)	49.28 ± 0.066	49.29 ± 0.071	0.951
**Cortical gray matter volume, %** (mean±SE)	37.63 ± 0.051	37.62 ± 0.055	0.883

Abbreviations: Magnetic resonance imaging (MRI); physical activity (PA); standard error (SE); p-value (p).
Adjustment model 1: age, age-squared, sex, time from exam to MRI, smoking, cohort, wear time, season of exam, residence in New England versus other, body mass index, hypertension, diabetes mellitus, cardiovascular disease
(not adjusting for light-intensity PA)

© 2019 Spartano NL et al. *JAMA Network Open.*

eTable 2. Relations of objectively measured physical activity to brain MRI measures in the full study sample (n=2354), with 2 adjustment models

Physical Activity Measure	Categories of Physical Activity	Model	**Total Cerebral Brain Volume**		**Total White Matter Volume**		**White Matter Hyperintensities Volume**		**Lateral Ventrical Volume**	
			**β ± SE**	**p**	**β ± SE**	**p**	**β ± SE**	p	**β ± SE**	**p**
Total Activity (Steps/d)	0-5000 steps (n=489)	2	0	Ref	0	Ref	0	Ref	0	Ref
		3	0	Ref	0	Ref	0	Ref	0	Ref
	5000-7500 steps (n=684)	2	0.09 ± 0.16	0.599	-0.09 ± 0.12	0.472	0.01 ± 0.06	0.847	**-0.12 ± 0.05**	**0.031**
		3	0.09 ± 0.16	0.591	-0.08 ± 0.13	0.511	0.02 ± 0.06	0.741	**-0.12 ± 0.06**	**0.038**
	7500-10000 steps (n=581)	2	0.29 ± 0.17	0.096	0.14 ± 0.13	0.288	0.009 ± 0.06	0.880	**-0.16 ± 0.06**	**0.006**
		3	0.29 ± 0.17	0.096	0.15 ± 0.13	0.246	0.02 ± 0.06	0.741	**-0.16 ± 0.06**	**0.006**
	10000+ steps (n=596)	2	**0.35 ± 0.18**	**0.045**	0.03 ± 0.13	0.849	-0.03 ± 0.06	0.683	**-0.14 ± 0.06**	**0.015**
		3	**0.37 ± 0.18**	**0.039**	0.04 ± 0.14	0.755	-0.01 ± 0.06	0.845	**-0.15 ± 0.06**	**0.015**
Light-intensity PA,* per hour increments		2	**0.22 ± 0.07**	**0.003**	0.05 ± 0.06	0.377	**0.07 ± 0.03**	**0.007**	**-0.05 ± 0.02**	**0.028**
		3	**0.21 ± 0.07**	**0.003**	0.05 ± 0.06	0.375	**0.07 ± 0.03**	**0.009**	**-0.06 ± 0.02**	**0.014**
MVPA,* min/d	0-10 min (n=602)	2	0	Ref	0	Ref	0	Ref	0	Ref
		3	0	Ref	0	Ref	0	Ref	0	Ref
	10-20 min (n=580)	2	0.29 ± 0.16	0.076	**0.25 ± 0.13**	**0.047**	-0.08 ± 0.06	0.192	-0.10 ± 0.06	0.066
		3	0.30 ± 0.17	0.071	0.25 ± 0.13	0.052	-0.07 ± 0.06	0.272	-0.10 ± 0.06	0.066
	20-30 min (n=421)	2	0.35 ± 0.18	0.057	0.11 ± 0.14	0.443	-0.07 ± 0.07	0.255	**-0.14 ± 0.06**	**0.029**
		3	0.34 ± 0.19	0.072	0.11 ± 0.14	0.456	-0.06 ± 0.07	0.399	**-0.13 ± 0.06**	**0.043**
	30+ min (n=751)	2	0.14 ± 0.17	0.390	0.01 ± 0.13	0.940	-0.10 ± 0.06	0.084	-0.07 ± 0.06	0.240
		3	0.15 ± 0.17	0.397	0.01 ± 0.13	0.919	-0.09 ± 0.06	0.160	-0.06 ± 0.06	0.296

Abbreviations: Magnetic resonance imaging (MRI); moderate to vigorous physical activity (MVPA); minutes (min); day (d) ; beta estimate (β); standard error (SE); p-value (p).

Adjustment Model 2: age, age-squared, sex, time from exam to MRI, smoking status, cohort, wear time, season of exam, residence in New England.

*Light-intensity PA models were additionally adjusted for MVPA categories (≥10min v. <10 min); and MVPA models were additionally adjusted for light-intensity PA.

Adjustment Model 3: Model 2 + body mass index, hypertension, diabetes mellitus, cardiovascular disease

© 2019 Spartano NL et al. *JAMA Network Open.*

eTable 3. Relations of objectively measured physical activity to total cerebral brain volume in participants aged 40-79 years (n=1922), by decade of age

Multivariable Regression Models in Full Sample, Total Cerebral Brain Volume	Age 40-49 (n=696)		Age 50-59 (n=602)		Age 60-69 (n=385)		Age 70-79 (n=239)	
	**β ± SE**	**p**	**β ± SE**	**p**	**β ± SE**	**p**	**β ± SE**	**p**
Total Activity (Steps/d) ≥7500 (n=957) v. <7500 (n=962)	0.10 ± 0.19	0.59	0.17 ± 0.20	0.39	0.37 ± 0.30	0.21	0.65 ± 0.45	0.15
Light Activity,* per hour increments	0.11 ± 0.12	0.35	**0.36 ± 0.12**	**0.003**	0.12 ± 0.20	0.56	0.27 ± 0.25	0.30
MVPA,* min/d ≥10min (n=1752) v. <10 min (n=602)	-0.14 ± 0.27	0.61	0.31 ± 0.24	0.19	0.47 ± 0.32	0.14	0.51 ± 0.44	0.25

Abbreviations: Magnetic resonance imaging (MRI); moderate to vigorous physical activity (MVPA); minutes (min); day (d); beta estimate (β); standard error (SE); p-value (p). The upper limits for age ranges are exclusive.
Model 2: age, age-squared, sex, time from exam to MRI, smoking status, cohort, wear time, season of exam, residence in New England
*Light Activity models were additionally adjusted for MVPA categories (≥10min v. <10 min); and MVPA models were additionally adjusted for light activity.

© 2019 Spartano NL et al. *JAMA Network Open.*

eTable 4. Relations of objectively measured physical activity to secondary brain MRI measures in the full study sample (n=2354), with 2 adjustment models

**Full Sample (n=2354)**	Categories of Physical Activity	Model	**Hippocampal Volume**		**Total Gray Matter Volume**		**Cortical Gray Matter Volume**	
			**β ± SE**	**p**	**β ± SE**	**p**	**β ± SE**	**p**
Total Activity (Steps/d)	0-5000 steps (n=489)	2	0	Ref	0	Ref	0	Ref
		3	0	Ref	0	Ref	0	Ref
	5000-7500 (n=684)	2	0.002 ± 0.003	0.482	0.13 ± 0.13	0.316	0.04 ± 0.10	0.671
		3	0.003 ± 0.003	0.228	0.09 ± 0.14	0.521	0.02 ± 0.11	0.869
	7500-10000 (n=581)	2	0.004 ± 0.003	0.142	0.26 ± 0.14	0.071	0.12 ± 0.11	0.277
		3	**0.006 ± 0.003**	**0.041**	0.19 ± 0.14	0.186	0.09 ± 0.11	0.436
	10000+ (n=596)	2	0.001 ± 0.003	0.680	0.17 ± 0.15	0.231	0.08 ± 0.11	0.482
		3	0.004 ± 0.003	0.201	0.11 ± 0.15	0.443	0.05 ± 0.12	0.652
Light-intensity PA,* per hour increments		2	0.001 ± 0.001	0.281	0.05 ± 0.06	0.402	0.02 ± 0.05	0.657
		3	0.001 ± 0.001	0.238	0.05 ± 0.06	0.400	0.02 ± 0.05	0.707
MVPA,* min/d	0-10 min (n=602)	2	0	Ref	0	Ref	0	Ref
		3	0	Ref	0	Ref	0	Ref
	10-20 min (n=580)	2	0.003 ± 0.003	0.229	0.09 ± 0.14	0.486	0.02 ± 0.10	0.831
		3	0.005 ± 0.003	0.098	0.06 ± 0.14	0.659	0.005 ± 0.11	0.960
	20-30 min (n=421)	2	-0.005 ± 0.003	0.118	0.10 ± 0.15	0.498	-0.02 ± 0.12	0.848
		3	-0.003 ± 0.003	0.346	0.03 ± 0.15	0.863	-0.06 ± 0.12	0.590
	30+ min (n=751)	2	-0.002 ± 0.003	0.585	0.05 ± 0.14	0.741	-0.004 ± 0.11	0.972
		3	0.0009 ± 0.003	0.765	-0.03 ± 0.14	0.829	-0.04 ± 0.11	0.723

Abbreviations: Magnetic resonance imaging (MRI); moderate to vigorous physical activity (MVPA); minutes (min); day (d) ; beta estimate (β); standard error (SE); p-value (p).

Adjustment Model 2: age, age-squared, sex, time from exam to MRI, smoking status, cohort, wear time, season of exam, residence in New England.

*Light-intensity PA models were additionally adjusted for MVPA categories (≥10min v. <10 min); and MVPA models were additionally adjusted for light-intensity PA.

Adjustment Model 3: Model 2 + body mass index, hypertension, diabetes mellitus, cardiovascular disease

© 2019 Spartano NL et al. *JAMA Network Open.*
